# Miniaturized Water-Jet Ultrasound Indentation System for Quantitative Assessment of Articular Cartilage Degeneration: A Validation Study

**DOI:** 10.1155/2020/2316369

**Published:** 2020-07-15

**Authors:** Yan-Ping Huang, Yong-Ping Zheng

**Affiliations:** ^1^School of Physics and Optoelectronic Engineering, Foshan University, Foshan, Guangdong, China; ^2^Department of Biomedical Engineering, Hong Kong Polytechnic University, Kowloon, Hong Kong, China

## Abstract

Osteoarthritis is a common joint disease affecting a large population especially the elderly where cartilage degeneration is one of its hallmark symptoms. There is a need to develop new devices and instruments for the early detection and treatment of cartilage degeneration. In this study, we describe the development of a miniaturized water-jet ultrasound indentation probe for this purpose. To evaluate the system, we applied it to characterize the degeneration of articular cartilage with the measurement of its morphologic, acoustic, and mechanical properties, using the enzymatic digestions of cartilage as a model of OA. Fifty cartilage samples were tested with 10 of them used for the reproducibility study and the other 40 for collagenase and trypsin digestions. Thickness, integrated reflection coefficient (IRC), effective stiffness, and energy dissipation ratio (EDR) were used to quantify the change of articular cartilage before and after degeneration. The measurement reproducibility as represented by the standardized coefficient of variation (SCV) was 2.6%, 10.2%, 11.5%, and 12.8% for thickness, IRC, stiffness, and EDR, respectively. A significant change of IRC, stiffness, and EDR was detected after degeneration by the designed probe (*p* < 0.05). There was also a significant difference of IRC, stiffness, and EDR between trypsin and collagenase digestions (*p* < 0.001). In conclusion, a miniaturized water-jet ultrasound indentation probe has been designed, which has been successfully used to detect and differentiate cartilage degeneration simulated by enzymatic digestions. This probe, with future development, can be potentially suitable for quantitative assessment of cartilage degeneration with an arthroscopic operation.

## 1. Introduction

Osteoarthritis (OA) is a common joint disease, which is prevalent in old populations [1, 2]. Treatment of OA causes a big economic burden to the society [3] and therefore, it is worth a great effort to find a cost-effective management strategy for this disease [4]. Cartilage is one of the hallmark tissues degraded in OA which are normally detected by clinical symptoms such as limited joint movement and a significantly narrowed joint space as seen in X-radiography. However, symptomatic detection is subjective rather than objective and results of plain radiography can easily be affected by the imaging procedure or joint positioning [5]. Furthermore, significant joint space narrowing is normally a sign of very late stage of articular cartilage degeneration [6], for which currently no effective treatment exists. Therefore, it is necessary to develop more sensitive methods, using facilities such as MRI [7] or ultrasound [8] to detect the early degradation of this tissue, when the disease is still reversible and treatment using disease-modifying OA drugs may be more effective [9].

Articular cartilage is a thin but complex tissue covering the bony joint end. Main functions of articular cartilage are to provide mechanical support and lubrication during the joint movement. Collagen fibrils and proteoglycans (PGs) are the two main macromolecules forming the extracellular matrix and significantly contribute to the mechanical properties of the articular cartilage [10]. While normal cartilage shows a smooth and shining appearance at its surface, early degeneration of articular cartilage may involve the fibrillation at the cartilage superficial layer which increases the surface roughness [11, 12]. More importantly, the change of tissue compositions such as collagens and aggrecans in early degeneration will also induce degradation of the mechanical properties including softening and change of viscosity [13]. Therefore, it is recognized that it is potential to detect the early degeneration of articular cartilage through a quantification of the mechanical properties as well as morphologic characteristics of this tissue [14–20].

Indentation is one of the most frequently used methods to measure the mechanical properties of soft tissues [21, 22]. There is a need to measure the tissue thickness as it is a critical parameter in analyzing the indentation data especially when it is comparable to the indenter size [22]. Ultrasound was purposely introduced in the indentation test because it can measure both deformation and thickness of the tested soft tissue simultaneously [23, 24]. It was coined ultrasound indentation and has also been used for the characterization of articular cartilage [8, 25, 26]. In ultrasound indentation of cartilage, the ultrasound can be used as an additional tool to measure its acoustic properties such as surface reflection, backscattering, and attenuation [8, 17, 27–29]. However, there is still limited flexibility to select the ultrasound transducer in ultrasound indentation as it will be used as an indenter as well, which has a specific shape requirement. The adoption of a water-jet ultrasound indentation avoids the use of a rigid indenter, which brings flexibility to the selection of ultrasound transducer [30, 31]. The workbench version of this system has been demonstrated to be useful for a multivariate characterization of the degeneration of articular cartilage using morphologic, acoustic, and mechanical parameters [32]. However, considering a minimally invasive operation as compared to traditional open surgeries [33] and to make this technique accessible for an *in vivo* measurement of diarthrodial cartilage, miniaturization of this system appropriate for arthroscopic operation is a must [34]. Therefore, in this study, we present some progress in this aspect with regard to a miniaturized water-jet ultrasound indentation system and its performance validation in quantifying the morphologic, acoustic, and mechanical properties of articular cartilage for the detection of cartilage degeneration.

## 2. Materials and Methods

### 2.1. Probe Construction and Data Collection System

**Figure 1:**
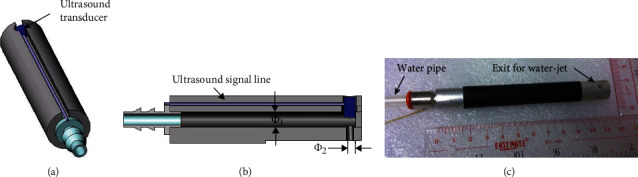
(a) A perspective view of the probe schematics, (b) A schematic view of its internal structure, and (c) a real picture of the constructed probe.

The miniaturized probe was constructed as shown in Figure 1. It mainly consisted of an aluminum rod of 12 mm in diameter and 11 cm in length. A central channel with *Φ*_1_ = 3 mm in diameter was excavated and a 10 MHz plane wave ultrasound transducer (XMS-310-B, Panametrics, Olympus NDT Inc., Waltham, MA, USA) with 3 mm in diameter was firmly installed at the tip. A channel with *Φ*_2_ = 2 mm in diameter was drilled on the opposite side of the transducer as the exit of water-jet, also serving as the passage of ultrasound signals. A water pipe is connected at one end of the aluminum rod for input of the water-jet.

**Figure 2:**
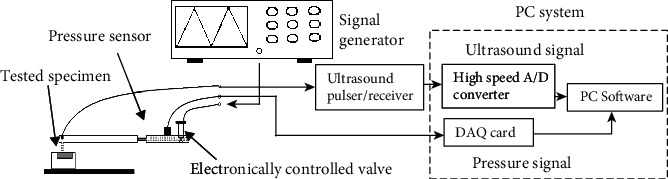
A flowchart of the data collection system for the miniaturized water-jet ultrasound indentation probe.

The data collection system incorporating the miniaturized probe is shown in Figure 2. On the control side, the pressure of water-jet was adjusted by an electronically controlled proportional hydraulic valve (2835-A-04, Christian Burkert GmbH & Co. KG, Ingelfingen, Germany). A signal generator was used to generate a cyclic sawtooth voltage signal to control the hydraulic valve. The ultrasound transducer was excited by an ultrasound pulser/receiver (Panametrics 5601A, Olympus NDT Inc., Waltham, MA, USA). On the signal collection side, this pulser/receiver also received the reflected ultrasound signal before it was transferred to a personal computer for digitization by a 500 MHz sampling frequency 8-bit A/D converter (CS8500, Gage Applied Technologies Inc., Lockport, IL, USA). Ultrasound signals were sampled in the A-line format with a fixed length of 4096 points. Water pressure was recorded by a pressure sensor (PMP 1400, GE Druck Ltd., Leicester, UK) with a maximum measurement capacity of 4 bars, and this signal was digitized by a 12-bit DAQ card (PCI 6024E, National Instruments Co., Austin, TX, USA). The ultrasound A-line signal and the pressure signal were synchronically sampled with a frame rate of 10 Hz and then saved for offline analysis. A software program written in Microsoft C++ was used for the data collection and the offline processing. The deformation during indentation was calculated by tracking the movement of the interface reflections from the upper surface of the tested articular cartilage using a cross-correlation algorithm, and the force of indentation was obtained from the water pressure after a calibration process [30].

### 2.2. Specimen Preparation and Experiment

**Figure 3:**
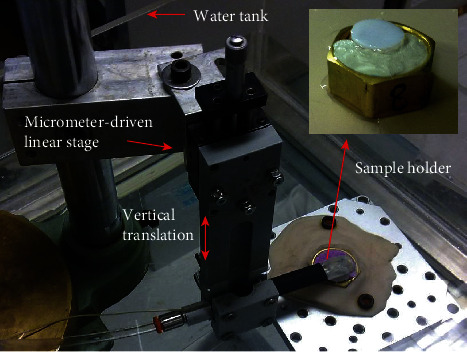
Testing setup and partial enlargement of the osteochondral cartilage sample holder.

Fresh mature bovine patellae with intact surface were obtained from a local market within six hours of sacrifice. For each patella, a cylindrical disk of 16 mm in diameter and approximately 15 mm in height was drilled from the upper lateral quadrant of the patella. Totally 50 osteochondral disks were prepared, among which 10 were used for the reproducibility study and the other 40 were used for the two enzymatic degeneration study. The osteochondral disk was fixed firmly in a sample holder with the cartilage surface adjusted at a horizontal plane using epoxy putty embedding (see the enlarged part of Figure 3). The osteochondral disk associated with the sample holder was then stored at -20°C in a refrigerator until the day of the test. Before the test, the disk was thawed in physiological saline for at least 2 hours under a room temperature of 24 ± 1°C. Two enzymes—collagenase and trypsin—were used to digest the cartilage samples to introduce a simulated degeneration of the cartilage, typically including the change of its collagen network and PGs. Collagenase is used to mainly digest the collagen network [35], and trypsin has the most significant effect on PG depletion with some minor effect on the collagen network [36]. The normal disks for testing (*n* = 40) were divided equally into two groups: collagenase (*n* = 20) and trypsin (*n* = 20) treatment groups. For the collagenase treatment group, disks were immersed in 30 U/ml collagenase solution (GIBCO, Invitrogen Corporation, Carlsbad, CA, USA) and kept in an incubator at 37°C for 24 h [17]. For the trypsin treatment group, disks were placed in 0.25% trypsin-ethylenediaminetetraacetic acid (EDTA) solution (GIBCO, Invitrogen Corporation, Carlsbad, CA, USA) and kept in the incubator at 37°C for 4 h [17]. After the digestion, the disks were rinsed and submerged in the physiologic saline solution for 1 hour before the retest.

During the test, the probe was installed in a horizontal direction with a maneuverable arm which could be translated in a vertical direction using a micrometer-driven linear stage (Figure 3). The probe could also be rotated along its long axis to adjust the acoustic beam direction so that a maximum signal could be obtained from the cartilage surface to ensure a perpendicularity of the incident ultrasound entering the cartilage. The sample holder with an embedded osteochondral disk was installed in a heavy base stage for a steady fixation during the test. The probe was just placed over the center of the osteochondral disk, and the distance between the probe end and the cartilage surface was set to be 1 mm. The base stage was immersed in a water tank during the measurement (Figure 3). The experiment was done as follows: firstly, the cartilage was measured for thickness and an acoustic parameter, i.e., the surface reflection. In detail, the ultrasound A-line signals reflected from the center of the disk were measured three times. Between two consequent tests, the sample was taken away, placed back with rough relocation, and the orientation of the probe was readjusted to find a maximally reflected signal for each measurement. After data collection for the morphologic and acoustic parameters, a cyclic water-jet indentation test was then conducted on the osteochondral disk. Totally, four cycles of loading and unloading of the cartilage were conducted with a cycle length of 10 s in one indentation test. The maximum pressure of the water-jet was approximately 330 kPa, which equaled to a maximal indentation force of 0.5 N through calibration. Three repeated tests were conducted for the water-jet indentation as described above by disk removal and relocation. Between any two consequent indentation tests, the sample was placed back into a physiological saline solution for recovery for at least half an hour [17, 37]. For reference, the osteochondral disk was also tested using a traditional rigid indentation under a standard mechanical testing machine (Instron 5569, Instron Co., Norwood, MA, USA) after the water-jet indentation. A stainless steel cylindrical indenter with a plane surface of 2 mm in diameter was used and similar indentation protocols were adopted in this test: a maximum indentation force of 0.6 N, an indentation speed of 1 mm/min, and four indentation cycles. The averaged value of those repeated tests was used to represent the properties of each disk. For the reproducibility test, six tests were conducted for each disk.

### 2.3. Parameter Extraction

The following four parameters, i.e., thickness, integrated reflection coefficient, stiffness, and energy dissipation ration were extracted from the indentation test, the details of which are described as follows.

#### 2.3.1. Thickness

**Figure 4:**
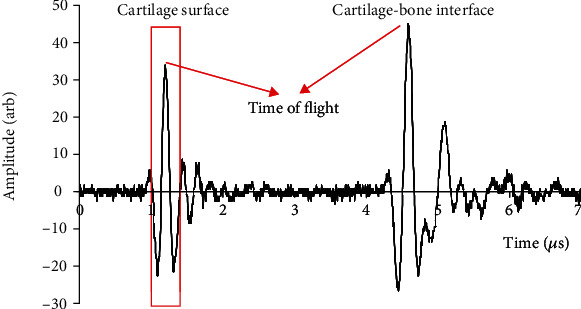
A typical ultrasound A-line signal from the osteochondral disk. The time of flight is used to calculate the cartilage thickness, and the signal in the rectangular window was used to calculate the integrated reflection coefficient (IRC). The calculated thickness is 2.49 mm, and IRC is -24.0 dB for this sample.

The thickness of cartilage was measured as the time of flight from the cartilage surface to the subchondral bone multiplied by the speed of sound. The time of flight was measured from the distance of the two peak echoes observed in the ultrasound A-line, which represent reflections from the water-cartilage interface and the cartilage-bone interface, respectively (Figure 4). A speed of sound value of 1610, 1595, and 1580 m/s was used in calculating the cartilage thickness in normal, trypsin-digested, and collagenase-digested conditions [17, 25].

#### 2.3.2. Integrated Reflection Coefficient (IRC)

IRC was used to represent the strength of ultrasound reflection from the cartilage surface, showing the difference of the acoustic impedance between the superficial layer of the cartilage and saline solution. The IRC was calculated after calibration based on the reference signal received from a stainless steel plate immersed in physiological saline solution at the same distance [17, 27, 38]. In detail, the surface reflection signal was obtained by applying a Hamming window of 140 points in length which was centered at the peak value (Figure 4). The windowed signal was then zero-padded to 1024 points, and FFT-transform was used to obtain its frequency spectrum *S*_*c*_(*f*, *d*) (*f* is frequency and *d* is the distance between transducer and cartilage surface). The frequency-dependent reflection coefficient was then obtained by:

(1) $$\begin{array}{c} R_{c}\left(f\right)=\frac{S_{c}\left(f,d\right)}{S_{r}\left(f,d\right)}, \end{array}$$

where *S*_*r*_(*f*, *d*) is the reference spectrum from a steel plate placed at the same distance with the cartilage surface. The distance *d* was neglected in *R*_*c*_(*f*) because the distance effect was cancelled after this calibration for the system-dependence. The reflection coefficient was then translated into a decibel unit and the integrated reflection coefficient was calculated as:

(2) $$\begin{array}{c} \text{IRC}=\frac{1}{∆f}\int_{∆f}R_{c}^{dB}\left(f\right)df, \end{array}$$

where *R*_*c*_^*dB*^(*f*) is the frequency-dependent reflection coefficient in the unit of dB and *∆f* is the -6 dB bandwidth of the ultrasound transducer, which was 4 to 18 MHz as measured in the current study.

#### 2.3.3. Stiffness and Energy Dissipation Ratio (EDR)

**Figure 5:**
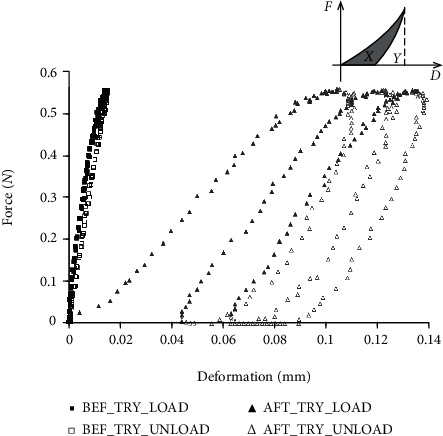
Two representative curves of force and deformation for the indentation test of a cartilage disk before and after trypsin digestion are shown. An averaged slope of force/deformation was extracted from each indentation curve to represent the elastic properties of the cartilage disk. The definition of energy dissipation ratio was schematically shown in the upper right corner of this figure: EDR = *X*/(*X* + *Y*), which represents the energy dissipated in the tissue during an indentation cycle. For this cartilage disk, the averaged slope of force/deformation is 41.6 N/mm and 7.8 N/mm and EDR is 12.5% and 61.4% before and after trypsin digestion, respectively. BEF: before, AFT: after, TRY: trypsin, LOAD: loading, UNLOAD: unloading.

The mechanical parameters of the cartilage were obtained from the indentation curve, which is typically shown in Figure 5 for a sample before and after trypsin digestion. For the elasticity, a stiffness coefficient was obtained using the following equation:

(3) $$\begin{array}{c} \text{SC}=\frac{F/A}{D/L_{0}}=\frac{F}{D}\cdot \frac{L_{0}}{A}=k\cdot \frac{L_{0}}{A}, \end{array}$$

where *k* = *F*/*D* in a unit of N/mm is the curve slope from a regression of the indentation force *F* and the deformation *D*, *L*_0_ is the initial thickness of cartilage as measured by ultrasound and *A* = *πd*^2/4^ is the indentation area, which is assumed to be a circle having the same size with the water-jet, i.e., *d* = 2 mm in diameter. The loading phase of the three indentation cycles from the second to the fourth was used to calculate the stiffness, regarding the first cycle as a preconditioning for the indentation test. During the calculation, a minimal indentation force of 0.05 N was used and the deformation was controlled to be within 3% of the initial thickness to guarantee a good linear relationship between stress and strain [39]. This was done in the offline data processing stage, where 0.05 N and 0.03*L_0_* were used to define the lower and upper bound of the region for calculation of stiffness coefficient. Considering cartilage is a viscoelastic, inhomogeneous, and anisotropic tissue, and the stiffness was obtained under specific experimental conditions, i.e., selected indentation speed at certain deformation range which reflected the mechanical properties of the whole cartilage layer, the stiffness was better called as “apparent stiffness”. For simplicity, “stiffness” is still used throughout this article. For the contact indentation test conducted in the mechanical testing machine, a Young's modulus *E* was calculated to represent the stiffness of the cartilage based on previous studies [22, 32]:

(4) $$\begin{array}{c} E=\frac{1-\nu^{2}}{2\pi a\kappa \left(a/h,\upsilon \right)}\cdot \frac{F}{D}, \end{array}$$

where *a* = 1 mm is the radius of the indenter, *ν* is the Poisson's ratio of the cartilage, *F*/*D* is defined as force/deformation ratio which has the same meaning with that of Equation (3), and *κ* is a scaling factor which is related to the aspect ratio *a*/*h* and the Poisson's ratio *ν*. A constant Poisson's ratio of 0.45 was used in the current study, assuming a large incompressibility of the cartilage [32]. The *κ* value can be obtained from a Table disclosed in the previous study [22]. To reduce the effect of cycles such as hysteresis typically shown in the degenerated cartilage, the stiffness or *E* was calculated for each indentation cycle separately and then averaged among cycles, which were processed in the same way with SC in the water-jet ultrasound indentation. The same protocols were used to calculate *F*/*D* for the contact indentation method as compared to the water-jet ultrasound indentation. For the viscous parameter, an energy dissipation ratio (EDR) was defined by calculating the percentage of energy dissipated in each indentation cycle. Energy dissipation is a general phenomenon induced by hysteresis which can be typically observed in a mechanical test of soft tissues [40, 41], which also exists for articular cartilage [42]. The loading and unloading curves in an indentation cycle form a closed area which represents the dissipated energy. If the closed area is *X* and the area under the unloading curve is *Y* (see upper right corner of Figure 5), then EDR is defined as:

(5) $$\begin{array}{c} \text{EDR}=\frac{X}{X+Y}. \end{array}$$

EDR is a percentage value calculated by a numerical integration based on the trapezoid rule. All the data points were used in each indentation cycle for calculating EDR. All the extractions of acoustic and mechanical parameters were conducted using scripts of Matlab (Mathworks Inc., Natick, MA, USA) using data recorded in the water-jet ultrasound and contact indentation system.

### 2.4. Statistical Analysis

For the reproducibility study, we used a standardized coefficient of variation (SCV) as an indicator. In detail, for sample *j*, the measurement coefficient of variation (CV) is

(6) $$\begin{array}{c} {\text{CV}}_{j}=\frac{{\text{SD}}_{j}}{\mu_{j}}, \end{array}$$

where *μ*_*j*_ is the mean and SD_*j*_ is the standard deviation of the six measurements on *j*. For all the samples, the global measurement CV is calculated as [43]:

(7) $$\begin{array}{c} \text{CV}=\frac{\sqrt{\sum_{j=1}^{m}{\text{SD}}_{j}^{2}/m}}{\sum_{j=1}^{m}\mu_{j}/m} \end{array}$$

where *m* = 10 represents the total number of cartilage disks. To compare the variation induced by measurement with that induced among samples without the effect of mean, SCV is defined as [44]:

(8) $$\begin{array}{c} \text{SCV}=\frac{\text{CV}\cdot \mu_{1\text{st}}}{4{\text{SD}}_{1\text{st}}}, \end{array}$$

where *μ*_1st_ and SD_1st_  represent the mean and standard deviation of the samples when only the first measurement of each sample was used for the calculation.

Statistical tests were performed to evaluate the change of tissue properties induced by enzyme digestions. The parameters before and after each enzyme treatment were compared using the paired-*t* test and comparison between the effects of trypsin and collagenase was conducted using the unpaired *t*-test. For comparison between the effects of trypsin and collagenase, a percentage value is calculated for the treatment effect using the following equation:

(9) $$\begin{array}{c} V_{R}=\frac{V_{\text{post}}-V_{\text{pre}}}{\left|V_{\text{pre}}\right|}\cdot 100\%, \end{array}$$

where *V*_*R*_ is the percentage change for each parameter *V* and the subscript indicates whether it is pre- or postdigestion value. The percentage change was calculated for the parameters of thickness, IRC, and stiffness. For EDR, a direct difference of between postdigestion and predigestion was used as it was already a percentage value. To compare the results of viscoelastic parameters with the reference indentation method, the Pearson correlation coefficient was calculated. A level of *p* < 0.05 was used to indicate a significant difference or a significant correlation. All the statistical tests were performed using the SPSS software (v25.0, SPSS Inc., Chicago, IL, USA).

### 2.5. Histology

After indentation tests, six normal cartilage samples from the reproducibility study, six samples from the trypsin-digested group, and six samples from the collagenase-digested group were randomly selected from each group for histological evaluation of proteoglycan content according to the protocol used in an earlier study [17]. After fixation, dehydration, and sectioning, the Safranin O plus fast green staining was employed to observe the change of PG in cartilage samples. After staining, the region stained with Safranin O red showed where PG existed while that with green color indicated the depletion of PG in the histological image [45].

## 3. Results

### 3.1. Reproducibility Study

The measurement SCV of the four parameters obtained from the miniaturized probe was 2.6% for thickness, 10.2% for IRC, 11.5% for stiffness, and 12.8% for EDR. Thickness measurement was the most highly reproducible, while that of IRC, stiffness, and EDR was similar (of ~10%). The SCV of measurement from the rigid indentation test was 6.5% and 10.0% for E and EDR, respectively. The reproducibility of the water-jet ultrasound indentation test was slightly lower than that of the mechanical testing from the rigid indentation test with respect to the two viscoelastic parameters.

### 3.2. Enzymatic Digestion Effect

The change of thickness and IRC before and after enzymatic digestions is shown in Table 1. There was no significant change of thickness after both the enzymatic digestions. For IRC, it significantly decreased after collagenase treatment (*p* < 0.001) while the treatment of trypsin had no significant effect (*p* > 0.05), which was consistent with previous studies [17, 25]. The change of IRC induced by trypsin was significantly larger than the collagenase (*p* < 0.001, Table 1).

The change of stiffness and EDR after enzymatic digestions is also listed in Table 1. The compressive stiffness of the cartilage significantly decreased after both the trypsin and collagenase digestions (both *p* < 0.001). The change of stiffness induced by trypsin treatment was significantly larger than that by collagenase treatment (-66.4% vs. -51.8%, *p* < 0.01). EDR was a small value in normal cartilage (~20%); however, it significantly increased (to ~60%) after the trypsin and collagenase digestion (both *p* < 0.001). This increase was significantly larger for the trypsin treatment than the collagenase treatment (40.6% vs. 32.9%, *p* < 0.01). The results showed that the current probe can be used to discriminate the degenerations of articular cartilage induced by different enzymes.

### 3.3. Comparisons with Reference Methods

**Figure 6:**
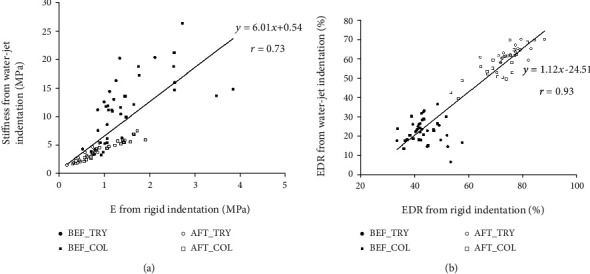
Correlation of results between the water-jet ultrasound indentation and the rigid indentation for (a) stiffness parameter (stiffness vs. E) and (b) EDR. Sources of the data points before or after trypsin or collagenase treatment are also indicated in the figure. Significant correlations of stiffness parameter and EDR are obtained between the two mechanical testing methods (both *p* < 0.001).

The results from the rigid indentation test are also included in Table 1. It was found that *E* significantly decreased after the two enzymatic digestions (both *p* < 0.001), with the degree of decrease by trypsin treatment being significantly larger than that by collagenase treatment (-49.7% vs. -24.6%, *p* < 0.001). For EDR, it significantly increased after both enzymatic digestions (both *p* < 0.001). The increase was slightly larger for the trypsin digestion than the collagenase treatment but did not reach a significant level (31.0% vs. 28.3%, *p* = 0.06). The correlation of results from the two mechanical testing methods was analyzed. There was a significantly positive correlation between stiffness from the water-jet indentation and *E* from the rigid indentation (*r* = 0.73, *p* < 0.001, Figure 6(a)). EDR measured from the two methods was also significantly correlated (*r* = 0.93, *p* < 0.001, Figure 6(b)).

**Figure 7:**
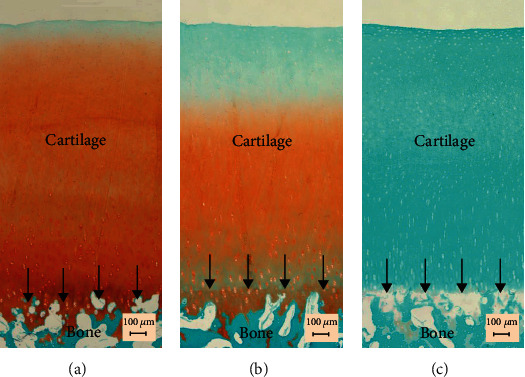
Typical histological pictures showing the Safranin O and fast green staining for the (a) normal (b) collagenase-digested and (c) trypsin-digested cartilage. It is typically observed that in collagenase-digested cartilage, there was partial loss of proteoglycans while they were completely depleted in trypsin-digested samples. Arrows indicate the cartilage-bone interfaces.

Typical histological results are shown in Figure 7. Based on observations of all the histological images, it was found that PG contents were intact in the normal cartilage. For the trypsin treatment, almost all PGs were digested while for the collagenase treatment, there was also partial PG loss, for which the reason was discussed in the following section.

## 4. Discussion

In the current study, the development of a miniaturized water-jet ultrasound indentation probe was presented and this probe was successfully applied to characterize the degeneration of articular cartilage *in vitro* using two enzymatic digestions as a model of material deterioration. A series of parameters indicating the morphologic, acoustic, and mechanical properties of the cartilage were successfully measured based on the single probe operation and could be used to probe the material change, and further, to differentiate the change caused by different mechanisms of degeneration. The advantages and limitations of this miniaturized probe and the current testing method, together with future development, are discussed as follows.

### 4.1. Construction of the Miniaturized Probe

Compared with the dimension of a previous water-jet indentation system [30, 32], there was a significant improvement for the currently developed probe, which can be more suitable for arthroscopic applications. We have successfully constructed the miniaturized probe thanks to the adoption of a small size ultrasound transducer and a compact design of water-jet passage. Although the diameter of the ultrasound transducer (3 mm) was slightly larger than that of the exit orifice (2 mm), experimental data showed that the design would not block the received ultrasound signal and the signal quality was not significantly affected, which could be used for quantitative analysis. The frequency of the ultrasound transducer, which was directly purchased from the commercial sensor market, was 10 MHz in the current study. Its resolution was still limited for probing small tissues such as cartilage. With the advancement of modern ultrasound technology, smaller transducers such as those used in the endoscopic applications [46] with a high-resolution cartilage imaging is also possible, by which more spatial information of the cartilage can be obtained such as the surface roughness [17]. Among them, the intravascular ultrasound (IVUS) with a very small profile has been demonstrated to be applicable for the characterization of cartilage degeneration [47–49] and is very potential for this purpose. On the other hand, water-jet was successfully applied as an indentation medium to compress the cartilage in this study. Water-jet technology has been among the choices of the armamentarium in medical applications such as lavage and surgery [50, 51]. In order to cut the tissue in surgical operations, water-jet with pressure as large as hundreds of bars has been applied with a small nozzle of ~100 *μ*m [51]. However, as the purpose of our water-jet is to indent rather than destroy the tissue, the pressure used in our study is much smaller and the used nozzle size is much larger than that used in surgical applications. Based on our experience, when the maximal pressure is beyond 550 kPa (5 bar) for the current probe, the water-jet becomes quite turbulent, which will greatly affect the acquisition of the ultrasound signal and make the signal void for quantitative analysis. Therefore, a maximal pressure of smaller than this value (about 330 kPa) was used in the current study. Specialized design may be necessary in future studies if a larger water pressure is needed to induce a certain amount of deformation of the tissue. The current entity of the probe is just one realization of the system design. There are also other possibilities for designing the water-jet ultrasound indentation probe and design parameters can be optimized such as the shape of the water-jet exit and the angle of water-jet to the cartilage surface during the operation, which warrants further investigations.

### 4.2. Cartilage Assessment Using the Miniaturized Probe

Based on the successful construction of the probe, we have reported some tests of a similar system using silicone phantoms [52]. In this study, we tested its performance on real cartilage samples using the enzymatic digestion as a model to simulate the degeneration of articular cartilage. Disruption of the collagen network and depletion of PG are the most significant changes of the extracellular matrix observed in degenerated articular cartilage in OA [53, 54]. Therefore, in this study, two specific enzymes, i.e., trypsin and collagenase were used to digest the two main components in cartilage to simulate the degeneration. The Safranin O staining showed that most of the PG was digested in the trypsin treatment. Only partial PG was digested in collagenase treated samples, which might be due to the porous structure formed after the collapse of the collagen network [17]. The histological study showed that enzymatic digestion could be truly used as a model to simulate the complete or partial breakdown of main cartilage components.

For the specific analysis, the reproducibility study showed comparable or even better reproducibility of the current system compared to our previous system in terms of SCV [17], and it was demonstrated that the quantitative parameters obtained from the miniaturized probe could be used to characterize the morphologic, acoustic, and mechanical properties of the cartilage and its change after degeneration. In detail, the cartilage thickness was calculated from two peak signals reflected from the two interfaces of the cartilage with the water and with the bone, respectively. The reflection from the surface of the cartilage comes directly from the difference of acoustic impedance of water and tissue. Therefore, it serves not only as a reference for the thickness measurement but also the acoustic impedance properties of the cartilage, which has been significantly changed after its compositional change, for example, in the collagenase digestion process. The thickness was found to have no significant change after two enzyme digestions. This was expected as an *in vitro* degeneration model was used in the current study. In living cartilage, the tissue may adapt to the disruption of extracellular matrix components in a symptom of thickness reduction. However, in an *in vitro* model, the tissue may lose the capability in adaption and therefore, immediately after the digestion, the change of thickness was neglectable [55, 56]. It is well known that the cartilage may become significantly thinner with the progression of OA grade [57]. However, it should be noted in the early stage of degeneration, the cartilage thickness may increase instead of decrease because of hypertrophic repair and swelling of the cartilage [58, 59]. Therefore, the measurement of thickness through the developed probe will be beneficial for the differentiation between the early or late stages of the cartilage degeneration. With regard to the amplitude of the acoustic reflection from the cartilage surface, the results of the IRC showed that the digestion using trypsin induced no significant effect while collagenase treatment significantly reduced this value [8]. Collagenase digestion caused the cleavage of the superficial collagen network and this reduced the acoustic impedance and increased the surface roughness of the cartilage, both of which would reduce the reflection of ultrasound from the surface [56, 60]. However, the PG content is small in the superficial layer of the cartilage [61] and change of the acoustic impedance and surface roughness is neglectable after the trypsin digestions. Accordingly, no significant change of surface reflection was observed from the group with trypsin digestion in this study. Therefore, the acoustic reflection from the cartilage surface could be practically used to differentiate between degenerations induced by cleavage of aggrecan or collagen fiber network.

**Figure 8:**
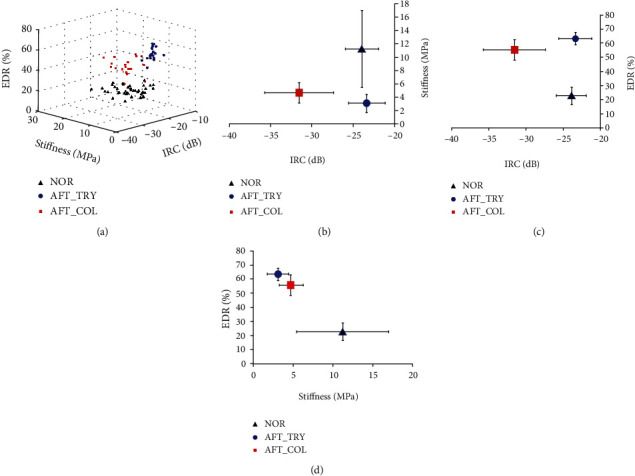
(a) A 3D scatter plot of three parameters IRC, stiffness, and EDR in normal and degenerated cartilage after trypsin and collagenase digestion. The corresponding projection of the 3D data sets into a 2-D plane in terms of mean and standard deviations of different groups are also plotted in (b) IRC vs. stiffness, (c) IRC vs. EDR, and (d) stiffness vs. EDR. Normal cartilages included those before the trypsin digestion and those before the collagenase digestion. NOR: normal, AFT_TRY: trypsin-digested samples, AFT-COL: collagenase-digested samples.

The results of the water-jet ultrasound indentation test on the cartilage showed that the cartilage stiffness significantly decreased and the energy dissipation during an indentation cycle significantly increased after the two enzymatic digestions. After trypsin digestion, the cartilage lost the PGs as shown in histology and the fixed charge density decreased, which significantly reduced the repulsive force during the compression and led to a smaller stiffness [62]. For the collagenase digestion, the breakdown of collagen fibers would form pores in the cartilage and some PGs would then easily move out of the extracellular matrix, both of which would also reduce the mechanical quality of the cartilage. However, it seemed that in the current model the effect of collagenase digestion was smaller than that of trypsin treatment in reducing the stiffness of the cartilage (Table 1), which might be due to that fact that most of PGs were depleted in the trypsin digestion while only partial PG and the superficial network were affected by the collagenase treatment. Viscosity is another important mechanical property of the biological soft tissue and in this study, the energy dissipation ratio (EDR) was used to investigate the change of cartilage viscosity before and after enzymatic digestions. From the indentation curve typically shown in Figure 5, the cartilage before enzymatic digestion behaved more like an elastic material with little hysteresis and preconditioning effect. However, the effects of hysteresis and preconditioning could be obviously observed after the digestion. It is well known that the hysteresis phenomenon shown in the mechanical testing of cartilage is mainly caused by the content of the interstitial fluid. EDR of cartilage test is shown to be loading rate dependent [42]. However, the same indentation speed was used in all the mechanical testing of the current study, and therefore the change of EDR after enzymatic digestion was not caused by the difference of loading rate. The change of EDR might come from the alteration of hydration or the solid/fluid interactions in the cartilage. After digestions, the water content in the cartilage might become higher [63] and the water might become easier to move with a higher permeability [64]. The combination of these changes together with the PG loss might make the tissue less capable of energy storage resulting in a larger value of EDR. The combination of the parameters measured in the current study could also be used to differentiate between normal and degenerated cartilage as well as between trypsin-digested and collagenase-digested cartilage.  Figure 8 shows a 3D scatter plot of the three parameters in normal and degenerated cartilage samples, where the corresponding 2-D plots using two parameters among them are also given. The normal group included the cartilage samples from both groups before enzymatic digestions. It can be easily observed from the figure that the three groups were clustered at different locations. As the standard deviation of stiffness in the normal cartilage was quite large, the combination of IRC and EDR might have the best discrimination between the three groups. Therefore, it was demonstrated that the probe could be used to discriminate between normal and degenerated cartilage and also between degenerated cartilages induced by the two different enzymes.

Analysis of the results obtained from the water-jet indentation test and from the rigid indentation test showed that the mechanical parameters were highly correlated and the trend of change was consistent (Figure 6 and Table 1). Therefore, using the rigid indentation as a conventionally validated reference method, the results demonstrated that the mechanical test using the miniaturized water-jet ultrasound indentation probe was effective to study the biomechanical properties of the articular cartilage. Both methods showed a significantly decreased stiffness and an increased EDR of the cartilage after enzymatic digestions. With respect to extent of change, it was found the decrease of Young's modulus measured from the rigid indentation induced by the two enzymatic digestions was smaller than that of stiffness coefficient from the water-jet indentation, especially for the collagenase treatment. With respect to EDR, its value from water-jet indentation was generally smaller than that of the rigid indentation. Both the differences might be originated from the two intrinsically different indentation methods. Interactions between the cartilage and indenter depend on the indenter material so that even with the same force level of indentation, the behavior of deformation may be quite different for two indentation methods. Further investigation using experimental or simulation methods is necessary to explain the differences of results from the two methods and extract intrinsic material parameters from the water-jet indentation.

### 4.3. Limitations of This Study and Future Research Directions

The current study has some limitations. Firstly, a simplistic model of enzymatic digestion was used to simulate the cartilage degeneration. The extent of component destruction may not be easy to be controlled to simulate an early degeneration of the cartilage using the enzymatic digestion model. Furthermore, the destruction to the cartilage using these enzymes is quite specific, and the model is quite simple with respect to the in reality very complicated etiology and pathology of the cartilage degeneration. Animal model of osteoarthritis such as anterior cruciate ligament transection or naturally degenerated cartilage samples may be adopted in future studies to demonstrate the utility of the developed probe. Secondly, a single-element ultrasound transducer was used in the current study so that it was cumbersome to realize the cartilage imaging. The imaging of the cartilage has several advantages for the qualitative and quantitative assessment including a comparison of lesions with histology [65], spatial averaging of calculated parameters such as IRC to improve measurement reliability, and studying the regional variation of the tissue properties such as surface roughness [17, 38]. Thirdly, a very simple model of the mechanical behavior of cartilage under indentation was adopted in the current study. We used a slope of the loading phase of the force-deformation curve to represent the elastic properties and an energy dissipation ratio to represent the viscous properties of the cartilage, under certain indentation test protocols. It is well known that cartilage is a complex multiple-phasic tissue with various characteristics of a typical biological soft tissue under mechanical test: inhomogeneous induced by hierarchical structure, anisotropic induced by fiber orientation, strain and strain-rate dependent induced by tissue fluid. How those more realistic and practical models such as biphasic or triphasic model [66, 67] together with a detailed analysis of the water-jet indentation to get intrinsic properties from the cartilage, which will be easily used in clinical practice, warrant further research. Lastly, although the current probe is a miniaturized version compared to our previous one, it is still not so practical for clinical trials. Our future work towards this direction will be to realize a practical arthroscopic channel-based probe with the incorporation of an intravascular ultrasound transducer [47]. Such work is currently ongoing [68].

## 5. Conclusions

In this study, we have fabricated a miniaturized water-jet ultrasound indentation probe and this probe was successfully applied to the bovine cartilage to characterize the degeneration induced by enzymatic digestions. Through the measurement using this miniaturized probe, a series of parameters including the morphologic, acoustic, and viscoelastic properties of the cartilage could be obtained and they could be used to differentiate between degenerations mainly induced by collagen network or proteoglycan destruction. This probe can be further designed using an arthroscopic channel with endoscopic ultrasound transducer such as an IVUS probe and then be potentially suitable for the detection of early degeneration of articular cartilage in osteoarthritis or for the quantitative assessment of cartilage repair *in vivo*.

## Figures and Tables

**Table 1:** Change of measured parameters before and after enzymatic digestions.

Parameters	Enzyme	Before treatment	After treatment	Change	
Thickness (mm)	Trypsin	1.96 ± 0.52	1.97 ± 0.54	−0.2 ± 4.4%	
	Collagenase	1.73 ± 0.37	1.73 ± 0.37	0.2 ± 3.6%	
IRC (dB)	Trypsin	−23.05 ± 1.80	−23.34 ± 2.22	−1.6 ± 9.1%	]^∗∗∗^
	Collagenase	−24.77 ± 1.92	−31.53 ± 4.15	−27.7 ± 16.5%^∗∗∗^	
Stiffness (MPa)	Trypsin	10.732 ± 5.154	3.119 ± 1.367	−66.4 ± 15.4%^∗∗∗^	]^∗∗^
	Collagenase	11.699 ± 6.426	4.679 ± 1.520	−51.8 ± 18.9%^∗∗∗^	
*E* (MPa)	Trypsin	1.298 ± 0.748	0.636 ± 0.351	−49.7 ± 17.8%^∗∗∗^	]^∗∗∗^
	Collagenase	1.590 ± 0.768	1.143 ± 0.420	−24.6 ± 13.3%^∗∗∗^	
EDR_WJ_ (%)	Trypsin	22.7 ± 6.9	63.2 ± 4.5	40.6 ± 9.2%^∗∗∗^	]^∗∗^
	Collagenase	22.7 ± 5.5	55.5 ± 7.5	32.9 ± 8.9%^∗∗∗^	
EDR_RI_ (%)	Trypsin	45.0 ± 5.8	75.9 ± 5.6	31.0 ± 3.9%^∗∗∗^	
	Collagenase	42.0 ± 4.5	70.3 ± 7.7	28.3 ± 6.4%^∗∗∗^	

Values are expressed as mean ± standard deviation (SD). IRC: integrated reflection coefficient, E: Young's modulus, EDR: energy dissipation ratio, WJ: water-jet, RI: Rigid indentation. Level of significance of change compared to pretreatment or comparison of changes between the two types of enzymatic digestion: ^∗∗^*p* < 0.001, ^∗∗∗^*p* < 0.001.

## Data Availability

The data of this study are available from the authors upon request.
